# Genome-wide Identification and Evolution of the *PP2C* Gene Family in Eight Rosaceae Species and Expression Analysis Under Stress in *Pyrus bretschneideri*

**DOI:** 10.3389/fgene.2021.770014

**Published:** 2021-11-11

**Authors:** Guoming Wang, Xun Sun, Zhihua Guo, Dirk Joldersma, Lei Guo, Xin Qiao, Kaijie Qi, Chao Gu, Shaoling Zhang

**Affiliations:** ^1^ State Key Laboratory of Crop Genetics and Germplasm Enhancement, Centre of Pear Engineering Technology Research, Nanjing Agricultural University, Nanjing, China; ^2^ Department of Cell Biology and Molecular Genetics, University of Maryland, College Park, MD, United States

**Keywords:** type 2C protein phosphatase, abscisic acid, pear (*Pyrus bretschneideri*), Rosaceae, abiotic stress

## Abstract

Type 2C protein phosphatase (PP2C) plays an essential role in abscisic acid (ABA) signaling transduction processes. In the current study, we identify 719 putative *PP2C* genes in eight Rosaceae species, including 118 in Chinese white pear, 110 in European pear, 73 in Japanese apricot, 128 in apple, 74 in peach, 65 in strawberry, 78 in sweet cherry, and 73 in black raspberry. Further, the phylogenetic analysis categorized *PbrPP2C* genes of Chinese white pear into twelve subgroups based on the phylogenic analysis. We observed that whole-genome duplication (WGD) and dispersed gene duplication (DSD) have expanded the Rosaceae *PP2C* family despite simultaneous purifying selection. Expression analysis finds that *PbrPP2C* genes have organ-specific functions. QRT-PCR validation of nine *PbrPP2C* genes of subgroup A indicates a role in ABA-mediated response to abiotic stress. Finally, we find that five *PbrPP2C* genes of subgroup A function in the nucleus. In summary, our research suggests that the *PP2C* family functions to modulate ABA signals and responds to abiotic stress.

## Introduction

Protein phosphorylation is a fundamental signal that regulates cellular processes, including growth factor responses, hormone responses, metabolic control, and developmental processes, and as with all signals, removal is as important as induction (den Hertog 1999; Kerk et al., 2002; Schweighofer et al., 2007). Protein kinases (PKs) phosphorylate serine (Ser), threonine (Thr), and tyrosine (Tyr) residues, whereas protein phosphatases (PPs) can reverse this action by removing the phosphate group (Luan, 1998; Wei et al., 2014). Therefore, the PPs are classified into three groups based on substrate specificity: protein Tyr phosphatases (PTPs), Ser/Thr phosphatases (STPs), and dual-specificity phosphatases (DSPTPs) (Cohen, 1989; Kerk et al., 2008). STPs are further divided into three subgroups: the phosphor-protein phosphatase (PPP), Mg^2+^- or Mn^2+^-dependent protein phosphatase (PPM), and aspartate-based protein phosphatases (Cohen 1989; Kerk et al., 2008). The PPP family covers PP1, PP2A, PP4, PP5, PP6, PP7, and PP2B, whereas the PPM family includes type 2C protein phosphatases (PP2Cs) and also pyruvate dehydrogenase phosphatases (Cohen, 1989; Kerk et al., 2008).

PP2Cs modulate and regulate protein kinase signaling cascades in archaea, bacteria, fungi, plants, and animals (Cao et al., 2016). In higher plants, *PP2C* genes were demonstrated to negatively regulate signaling pathways by opposing specific protein kinases (Tähtiharju and Palva, 2001; Yoshida et al., 2006). In *Arabidopsis*, 76 *PP2C* genes have been identified and categorized into ten groups (A–J), with the remaining six being uncategorized. Several *PP2C* genes from subgroup A have been verified as factors in ABA signaling (Hirayama and Umezawa, 2010). AP2C1 of subgroup B interacts with MPK4 or MPK6 to suppress MAPK activates in response to wounding and pathogen stresses (Schweighofer et al., 2007). POL or PLL1 of PP2C of subgroup C interacts with the receptor kinase CLV1 to regulate flower development and maintain stem cell polarity (Song et al., 2008; Gagne and Clark, 2010). AtPP2C6-6 of PP2C of subgroup E interacts with histone acetyl transferase AtGCN5 to modulate stomatal signaling (Servet et al., 2008). WIN2 of PP2C of subgroup F interacts with the bacterial effector HopW1-1 to induce stress response (Lee et al., 2008). Likewise, KAPP of unclustered PP2Cs interacts with RLKs to regulate plant immunity responses and hormonal signaling (Gomez-Gomez et al., 2001).

*PP2Cs* regulate plant development in both biotic and abiotic stress conditions (Sugimoto et al., 2014; Singh et al., 2016). At the molecular level, the *PP2C* function reflects its role in modulating signals transmitted by abscisic acid (ABA) (Merlot et al., 2001; Yoshida et al., 2006). In the ABA signaling pathway, PP2Cs can inactivate SnRK2 via dephosphorylation, and this inactivation was inhibited by ABA receptors (PYR/PYL/RCRA) (Soon et al., 2012). In *Arabidopsis,* proteins encoded by *PP2CA, ABI1,* and *ABI2* function in tolerance to exposure to salt, drought, and freezing (Strizhov et al., 1997; Merlot et al., 2001), and proteins encoded by *HAB1*, *HAB2,* and *AHG1* negatively regulate SnRK2 kinases required for ABA signaling (Saez et al., 2004; Saez et al., 2008; Umezawa et al., 2009). In *Fagus sylvatica*, *e*ctopic expression of *FsPP2C1* in *Arabidopsis* resulted in ABA insensitivity during seed germination (Gonzalez-Garcia et al., 2003), but ectopic expression of *FsPP2C2* in *Arabidopsis* resulted in enhanced ABA sensitivity and tolerance of abiotic stress in seeds (Reyes et al., 2006). This link between PP2C proteins and ABA signaling is ancient. In moss, *PpABI1A* and *PpABI1B* of subgroup A of *PP2C* function in drought tolerance via downregulation of ABA signaling (Komatsu et al., 2013), and in maize, *ZmPP2C-A10* also regulates drought stress tolerance (Xiang et al., 2017). Taken together, *PP2C* genes in subgroup A have been demonstrated to play key roles in plant development and environmental stresses.

In this study, we identify 719 *PP2C* genes from eight Rosaceae species. At the genomic level, we analyze the expression and phylogeny of the 118 *PP2C* genes found in Chinese white pear and analyze its evolution. At the protein level, we describe protein features and functions, domains of expression, and subcellular localization. Our findings set a foundation to understand the function of *PbrPP2C* genes in mediating responses to various stress conditions in a commercially important family of higher plants.

## Materials and Methods

### Sequence Retrieval Resources and Identification of Type 2C Protein Phosphatase Genes

For identification of *PP2C* genes in pear and other Rosaceae species, the HMM (hidden Markov odel) files were constructed by downloading the seed file PP2C (PF00481) domains from Pfam (http://pfam.xfam.org/) and were searched against the local protein databases using HMMER3 (Finn et al., 2015). The protein sequences of candidate *PP2C* genes were validated by using Interproscan 63.0 (http://www.ebi.ac.uk/InterProScan/) and Pfam (http://pfam.xfam.org/). The methods of screening and identification were identical to a previous report (Qanmber et al., 2019a; Qanmber et al., 2019b). *Arabidopsis PP2C* genes were downloaded from TAIR (http://www.arabidopsis.org/). Chinese white pear (*Pyrus bretschneideri*) and Japanese apricot’s (*Prunus mume*) genome sequences were obtained from the Pear Genome Project (http://peargenome.njau.edu.cn/) and *Prunus mume* Genome Project (http://prunusmumegenome.bjfu.edu.cn/index.jsp), respectively. The genome sequences of European pear (*Pyrus communis*), apple (*Malus domestica*), peach (*Prunus persica*), strawberry (*Fragaria vesca*), black raspberry (*Rubus occidentalis*), and sweet cherry (*Prunus avium*) were collected from the Genome database for Rosaceae (GDR) (http://www.rosaceae.org). The obtained PP2C protein sequences were screened for the PP2C catalytic domain by using the Pfam website (https://pfam.xfam.org/).

### Phylogenetic, Exon–Intron Structure, and Protein Motif Analysis

The full length protein sequences of *PP2C* were used to perform multiple sequence alignment, and the phylogenetic tree was performed using MEGA7.0 (Kumar et al., 2016) with the maximum likelihood method (ML), a bootstrap of 1,000 replications, and the Jones–Taylor–Thornton (JTT) model. The exon–intron organization of *PbrPP2C* genes was analyzed using CDSs and genomic DNA sequences using the Gene Structure Display Server (GSDS: http://gsds.cbi.pku.edu.cn/). Motif Elicitation (MEME: http://meme.sdsc.edu/meme/itro.html) was performed to identify conserved motifs of PbrPP2C proteins, with the maximum number of motifs = 20.

### *Cis*-Element Predictions of *Pbr*PP2C

All the *PbrPP2C* promoter sequences (selected as 2000 upstream bp) were downloaded from the Pear Genome Project (http://peargenome.njau.edu.cn/). The *cis*-regulatory elements of *PbrPP2C* were identified by the PlantCARE database (http://bioinformatics.psb.ugent.be/webtools/plantcare/html/).

### Chromosomal Locations, Synteny, and *Ka*/*Ks* Analysis of Type 2C Protein Phosphatase Genes

The chromosomal location information of the *PP2C* family genes was obtained from the genome annotation files. The analysis of synteny among eight Rosaceae genomes was performed by PGDD (http://chibba.agtec.uga.edu/duplication/) (Lee et al., 2013). BLASTP was carried out to identify multiple alignments of protein sequences (e-value < 10–5, top 5 matches) in the eight Rosaceae species. Then, MCScanX was carried out to produce orthologous gene pairs of *PP2C* within each Rosaceae species (Wang et al., 2012). Segmental/whole-genome duplication (WGD), tandem duplication (TD), proximal duplication (PD), transposed duplication (TRD), and dispersed duplication (DSD) in the *PP2C* gene family were identified by using the tools in the MCScanX package (Qiao et al., 2019). Localization and duplicate gene pairs of the *PP2C* genes were visualized using TB tools software (Chen et al., 2020). The values of *Ka* (non-synonymous substitutions) and *Ks* (synonymous substitutions) were calculated using *KaKs*_Calculator 2.0 with default parameters, and the *Ka*/*Ks* ratio was based on a model-averaged method (Wang et al., 2010).

### Transcriptome Expression Pattern Analysis in Different Tissues and in Different Ages of Pear Fruit

Previously published and unpublished dynamic RNA-seq data were used to analyze *PP2C* gene expression in different tissues of pear (Qiao et al., 2018; Li et al., 2019), including the pollen, seed, sepal, petal, ovary, bud, stem, leaf, and fruit. The raw RNA-seq reads were cleaned by removing low-quality reads (quality score <15), poly (A/T) tails, and adapter sequences. HISAT2 and feature counts were performed to align clean reads to the reference genome and estimate transcript abundance levels (Liao et al., 2014; Kim et al., 2015). Finally, the values of fragments per kilobase million (FPKM) were used to indicate the expression levels of *PP2C* genes. The heatmap of *PP2C* gene expression was visualized using TB tools software (Chen et al., 2020).

### Plant Material and Treatment

“Cuiguan” pear (*Pyrus pyrifolia* Nakai) seeds were collected from the pear germplasm orchard of the Pear Engineering Technology Research Center of Nanjing Agricultural University in Nanjing, China. Seedlings were grown for 5 weeks in a growth chamber, with a photoperiod of 16/8 h and a temperature of 25 ± 1°C. The seedlings were irrigated with 200 mM NaCl and 20% PEG 6000 for salinity and drought abiotic stress, respectively. Seedling leaves were sprayed with 100 μM ABA for ABA treatment. Seedlings were subjected to temperatures of 4°C and 37°C for low and high temperature stress, respectively. The leaves of treated seedling were collected at 0, 6, 12 and 24 h, respectively. The collected samples were quickly frozen in liquid nitrogen and stored at −80°C until further use.

### Quantitative Real-Time PCR Analysis

Total RNA was extracted using RNAprep Pure Plant Kit (Tiangen, Beijing, China). The extracted total RNAs were subjected to the first-strand cDNA using TransScript One-Step gDNA Removal and cDNA synthesis Supermix (TransGen, Beijing, China). The primers of 9 *PP2C* genes were designed using Primer Premier 6.0, and the tubulin gene of pear was used as the reference gene. All the primer sequences are listed in Supplementary Table S5. QRT-PCR was carried out in LightCycler 480 SYBRGREEN I Master (Roche, United States), and the reaction mixture and cycling program were identical to those of a previous report (Hao et al., 2018). All of the analyses were carried out with three independent biological replicates. The genes expression levels were calculated using the 2^−ΔΔCt^ method (Livak and Schmittgen, 2001).

### Subcellular Localization

For the subcellular localization analysis of PbrPP2Cs, the CDS sequences without the termination codon were amplified and cloned into pCAMBIA1300-35S: CDS-GFP vector. Primers used for cloning are listed in Supplementary Table S5. The recombinant plasmids and the control plasmid were transformed into 30-day-old tobacco (*Nicotiana benthamiana*) leaves according to the published protocol (Sparkes et al., 2006). Fluorescence was imaged using a confocal microscope LSM780 (Zeiss LSM 780, Germany).

## Results

### Identification, Characteristics, and Phylogenetic Relationship of Type 2C Protein Phosphatase Genes

A total of 719 putative *PP2C* genes were identified in eight Rosaceae species: 118 in Chinese white pear, 110 in European pear, 73 in Japanese apricot, 128 in apple, 74 in peach, 65 in strawberry (Haider et al., 2019), 78 in sweet cherry, and 73 in black raspberry (Supplementary Table S1). 118 putative *PP2C* genes of pear were arranged as *PbrPP2C1* to *PbrPP2C118* based on phylogenic analysis and the relative position of *Arabidopsis* orthologs. In addition, some members of the *PbrPP2C* gene family have two alternative splice variants, including *Pbr012020*, *Pbr019958*, *Pbr019984*, *Pbr022419,* and *Pbr031084* (Supplementary Table S1). Splicing variants played a crucial role in the posttranscriptional regulatory mechanism that modulates transcriptome and proteome diversity, such as alternative splicing of *PpDAM1* was important in the pear flower bud dormancy process (Li et al., 2021). The lengths of *PP2C* gene sequences ranged from 203 bp to 21,156 bp. Moreover, the protein molecular weights were 7.5–243 kDa, and the theoretical isoelectric point was from 3.9 to 10.49. The *PP2C* gene ID and the characteristics are shown in Supplementary Table S1.

**FIGURE 1:**
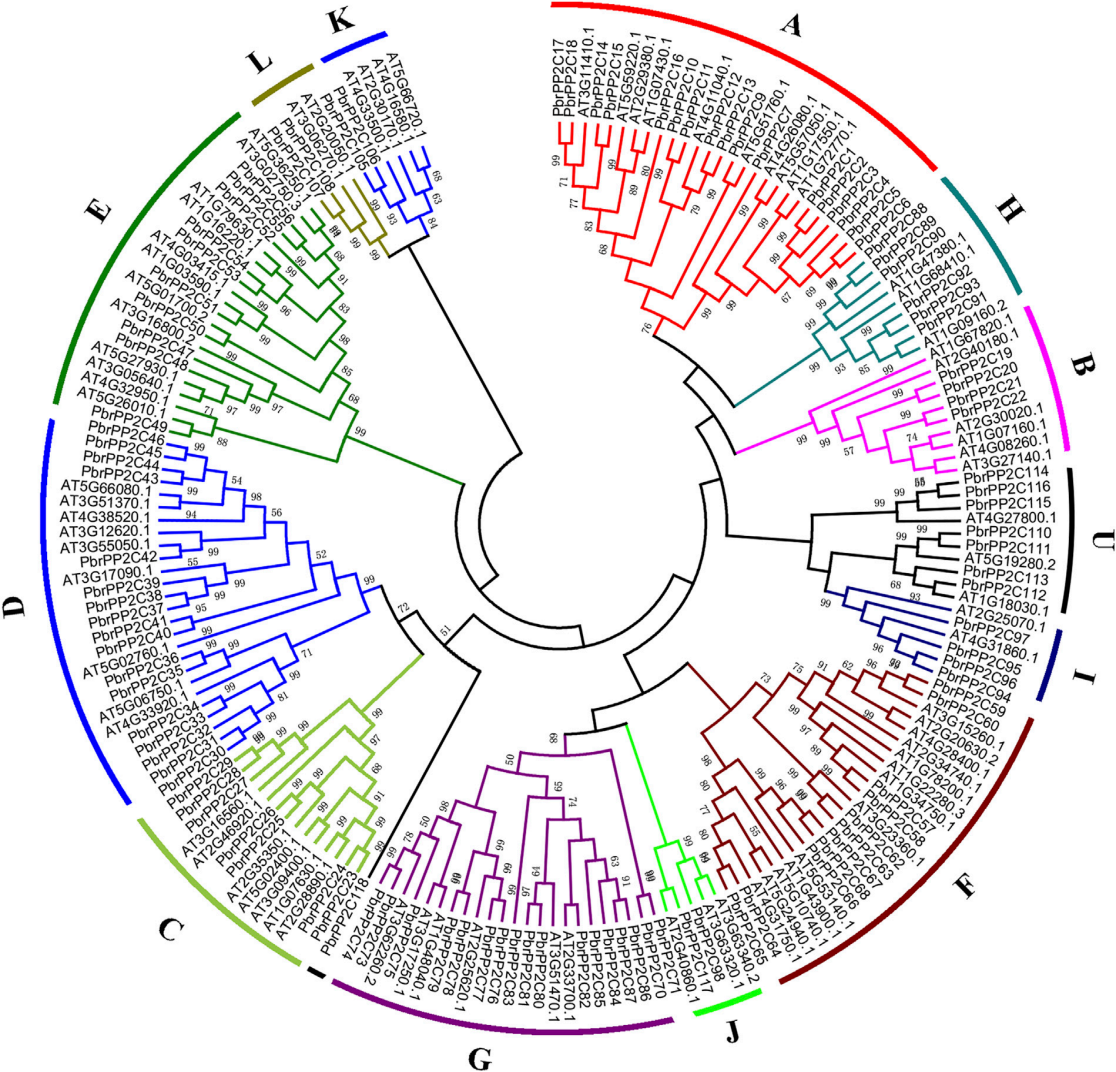
Phylogenetic analysis of the *PbrPP2C* family in both Chinese white pear and *Arabidopsis*. Phylogenetic tree was constructed using the maximum likelihood method.

To gain insights into the phylogenetic relationship of the *PP2C* genes in pear, a phylogenetic tree was constructed using MEGA7.0 by adopting the maximum likelihood method (ML) based on multiple sequence alignments of 80 *Arabidopsis PP2C* genes obtained from a previous study (Xue et al., 2008) and 118 pear *PbrPP2C* genes (Figure 1). This analysis divided 118 *PbrPP2C* genes into twelve subgroups: subgroups A–L and one unclassified subgroup U (Figure 1 and Supplementary Figure S1). Subgroups A, D, and G contain 18, 17, and 17 genes, respectively. Subgroup J, K, and L contain less.

In addition, the conserved motif analysis of *PbrPP2C* genes supported the phylogenetic analysis and classification (Supplementary Figure S1). A total of 20 motifs were identified in all the *PbrPP2C* family members. The PP2C proteins of each subgroup contained similar motifs. Motifs 1 and 5 were detected in almost all of the *PbrPP2C* genes, while Motif 4 was specific to subgroups C and D (Supplementary Figure S1). Most members of *PbrPP2C* subgroups contained more than 10 motifs, while a few members had 2–4 motifs, such as *PbrPP2C16*, *PbrPP2C71, PbrPP2C85, PbrPP2C113,* and *PbrPP2C118.* Different subgroups contain their own specific motifs that may lead to the functional divergence of each subgroup. To better understand the structures of *PbrPP2C* genes in pear, exon–intron organizations were compared among different subgroups (Supplementary Figure S1). The number of exons in the *PbrPP2C* family members varied from 1 to 21, and 37 genes were annotated in the 3′or 5′UTR region. Interestingly, *PbrPP2C* genes in the same subgroup show more or less similar exon–intron structures (Supplementary Figure S1).

### Chromosome Location and Collinearity Analysis of the Type 2C Protein Phosphatase Gene Family

**FIGURE 2:**
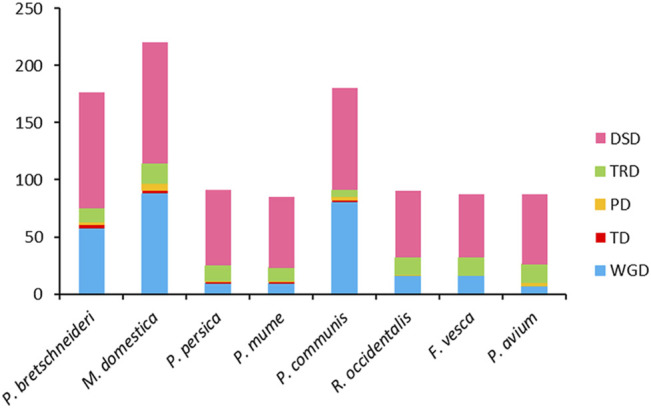
Number of *PP2C* gene pairs derived from different modes of gene duplication in pear and seven other Rosaceae species. *X*-axis represents the species. *Y*-axis represents the number of duplicated gene pairs. WGD: whole-genome duplication, TD: tandem duplication, PD: proximal duplication, TRD: transposed duplication, and DSD: dispersed duplication.

To explore the contribution of different gene duplication modes to the expansion and evolution of *PP2C* genes in eight Rosaceae species, a comparative analysis of gene duplication was performed in each genome (Figure 2, Supplementary Table S2). 1,014 duplicated gene pairs were found in the *PP2C* family members and were assigned to five duplication modes of WGD, PD, TD, TRD, or DSD. WGD is responsible for 8.2–44.4% of *PP2C* gene pairs in the investigated species. Consistent with lineage-specific duplications, Chinese white pear (32.4%), European pear (44.4%), and apple (40.0%) exhibit a relatively high proportion of WGD-derived *PP2C* genes. DSD accounts for the highest number of derived genes (48.2–72.9%), but TD (0–2.4%) and PD (0–2.7%) were observed with low frequency. TRD (3.3–18.4%) of *PP2C* gene pairs shows a high frequency in each of the Rosaceae species, which contributed to the formation of the *PP2C* gene clusters observed. The results are consistent with the inference that different gene duplication modes of *PP2C* gene pairs may have led to the neofunctionalization of ancestral genes.

**FIGURE 3:**
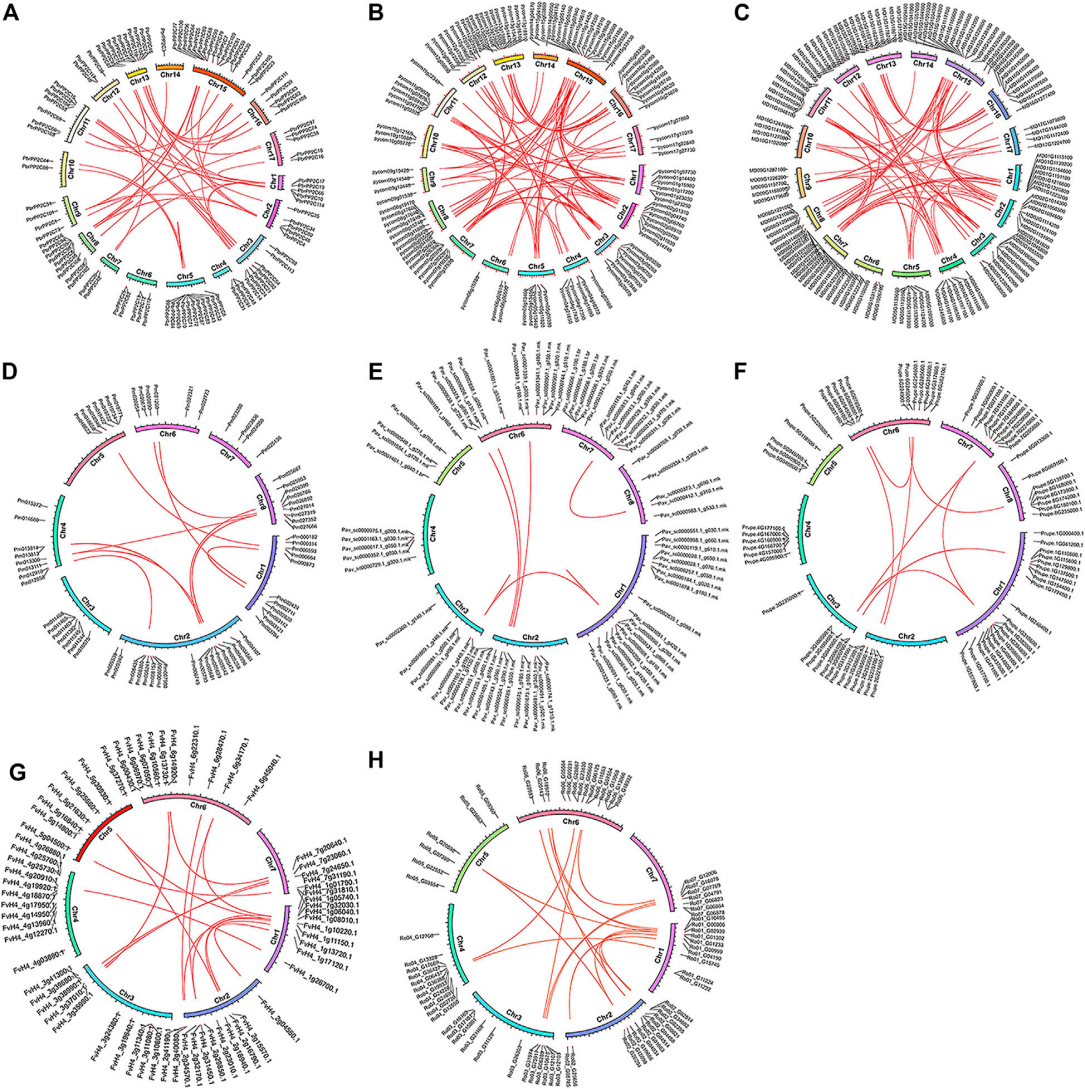
Gene location and collinearity analysis of the *PP2C* gene family. **(A)** Chinese white pear; **(B)** European pear; **(C)** apple; **(D)** Japanese apricot; **(E)** sweet cherry; **(F)** peach; **(G)** strawberry; **(H)** black raspberry. Genes were located on different chromosomes. Red lines represent the syntenic gene pairs.

We located the 719 *PP2C* genes on the chromosomes of each species based on genome annotations. For Chinese white pear, 118 of *PbrPP2C* genes were anchored onto all the 17 chromosomes and scaffolds, with the maximum number of genes detected on Chr 15 (17), followed by 11 genes on Chr 5. However, there was no correlation between the number of genes and the length of chromosomes, and the genes were randomly distributed on each chromosome (Figure 3A, Supplementary Table S2). *PP2C* genes were also found to be randomly distributed in the other Rosaceae species’ genomes. We identified 274 collinearity gene pairs, including 59 pairs in Chinese white pear, 16 pairs in strawberry, 78 pairs in apple, 80 pairs in European pear, 10 pairs in Japanese apricot, 8 pairs in peach, 16 pairs in black raspberry, and 7 pairs in sweet cherry (Figure 3, Supplementary Table S2). A large number of collinearity gene pairs were identified in Chinese white pear, European pear, and apple species.

### *Ka* and *Ks* Substitutions per Site and *Ka*/*Ks* Analysis for Type 2C Protein Phosphatase Family Genes

**FIGURE 4:**
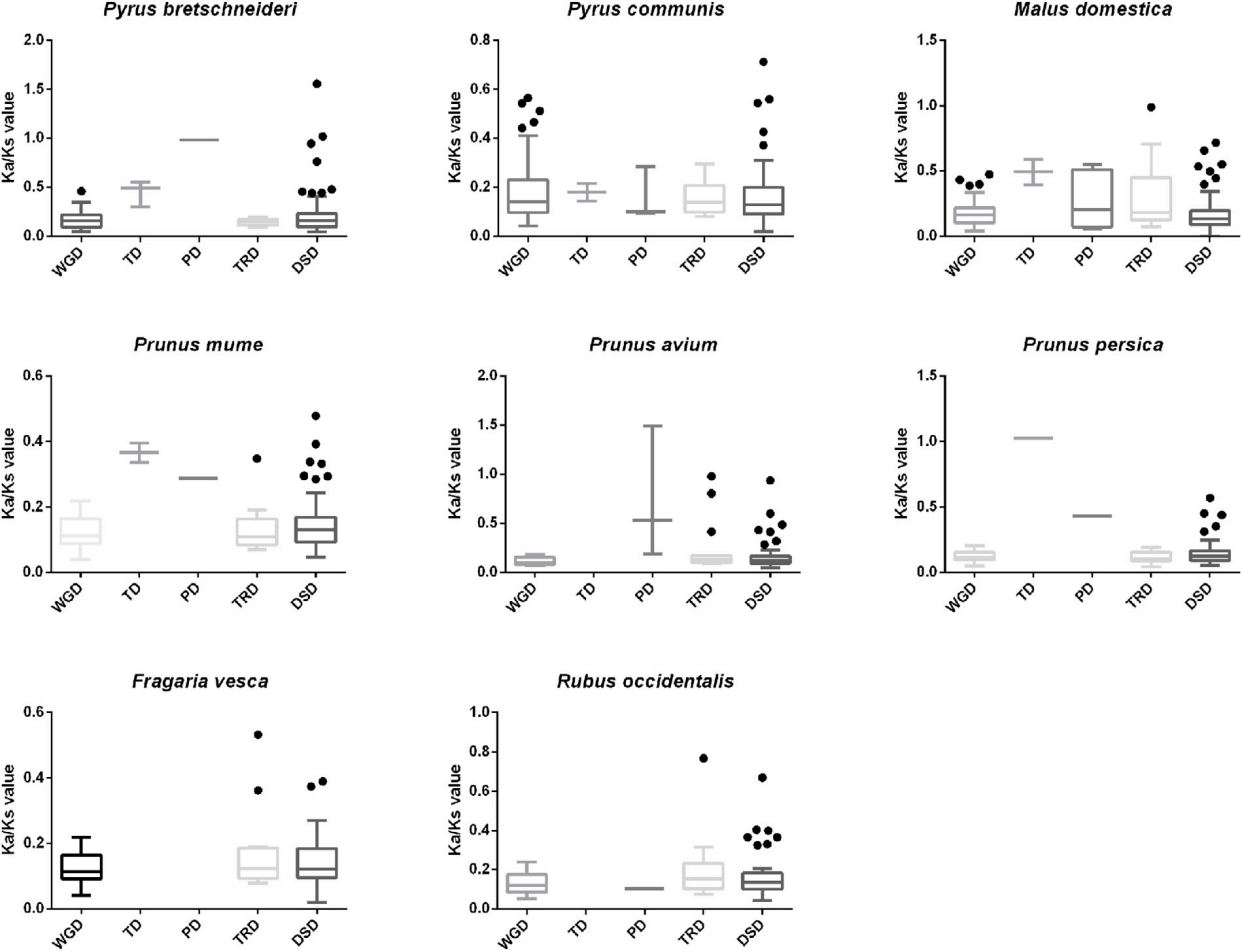
Ka/Ks distribution of eight Rosaceae species. Ka/Ks values were analyzed using coding sequences. *X*-axis represents five different duplication categories. *Y*-axis indicates the Ka/Ks ratio. T-boxplot was also constructed using prism 6.0.

The *Ks* value has been widely used to estimate the evolutionary stage of WGD events (Qiao et al., 2015). The mean *Ks* values of WGD-derived gene pairs in apple, European pear, Chinese white pear, Japanese apricot, black raspberry, sweet cherry, strawberry, and peach were 1.70, 1.90, 2.21, 2.40, 2.57, 2.907, 2.93, and 3.48, respectively (Supplementary Table S3). The lower *Ks* values of WGD-derived gene pairs in apple and European pear suggested that they were duplicated and retained from more recent WGD events, while sweet cherry, strawberry and peach were derived from more ancient WGD events. The *Ka*/*Ks* ratio is usually used to measure the magnitude and direction of selection pressure, and the *Ka*/*Ks* value refers to selection type: >1 indicate positive selection and <1 indicates purifying selection (Yang 2007). Purifying selection can remove deleterious mutations, and positive selection can induce favorable mutations (Starr et al., 2003). Here, the *Ka*/*Ks* values of *PP2C* orthologous gene pairs were calculated among eight Rosaceae species (Figure 4). The *Ka*/*Ks* values of duplicated gene pairs in European pear, Japanese apricot, black raspberry, and strawberry were less than 1, suggesting that *PP2C* genes evolved under strong purifying selection. Several gene pairs with higher *Ka*/*Ks* ratios were identified in apple, Chinese white pear, sweet cherry, and peach, suggesting that these genes may have a complicated evolutionary history. For Chinese white pear, the mean *Ka*/*Ks* ratios of WGD TD, PD, TRD, and DSD, were 0.17, 0.45, 0.98, 0.14, and 0.21, respectively (Figure 4). TD and PD had higher *Ka*/*Ks* ratios compared with other molds of duplicated gene pairs, suggesting that they evolved at a higher rate than the other gene pairs.

### Analysis of Putative Regulatory *Cis*-elements of the Type 2C Protein Phosphatase Gene Family

The *cis*-elements in promoter regions are closely related to gene transcription, and they play a critical role in plant signal transduction by interacting with their cognate transcription factor. Therefore, to better understand the function of *PbrPP2C* genes, 2.0 kb upstream promoter sequences of *PbrPP2C* genes were downloaded from the Pear Genome Database and analyzed by using PlantCARE. Some common *cis*-regulatory elements are briefly summarized and listed in Supplementary Table S6, such as ABRE was involved in ABA responsiveness, ERE was involved in ethylene responsiveness, MBS was the MYB binding site involved in drought-inducibility, and DRE was involved in adverse stress. The result showed that various *cis*-elements were related to plant hormones, light, abiotic stress, essential element, enhancer, circadian factors, and other regulatory stress responses (Supplementary Table S6). Consequently, various conserved *cis*-regulatory elements of PbrPP2C genes were crucial in mediating responses to various stress-related hormones or adverse biotic–abiotic stresses.

### Expression Profiling of the Type 2C Protein Phosphatase Gene Family in Different Tissues of Pear

**FIGURE 5:**
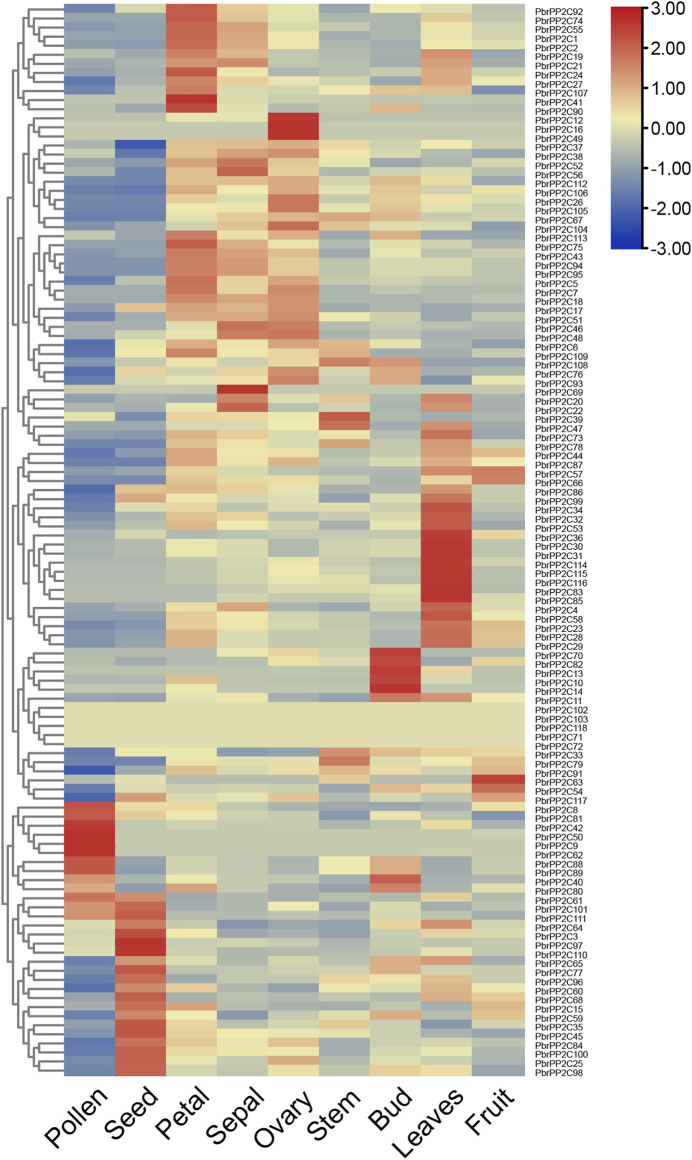
Heatmap of expression profiles (FPKM) for *PP2C* in the nine various tissues of pear (pollen, seed, petal, sepal, ovary, stem, bud, leaf, and fruit). Expression levels are indicated by the color bar.

To investigate the expression patterns of *PbrPP2C* family genes in different pear tissues, a heatmap was constructed using previously published RNA-seq data including matured pollen, seed, petal, sepal, ovary, stem, bud, leaf, and fruit (Figure 5 and Supplementary Table S4). Most *PbrPP2C* genes displayed a very broad expression range, and several *PbrPP2C* genes showed expressional activation in at least three or more tissues. Five genes (*PbrPP2C71*, *PbrPP2C72*, *PbrPP2C71*, *PbrPP2C102*, *PbrPP2C103,* and *PbrPP2C118*) exhibited very low or no expression. Eighteen *PbrPP2C* genes were found to be highly expressed preferentially in leaves, and five were highly expressed in buds. We identified three genes with ovary-specific expression (*PbrPP2C12*, *PbrPP2C16,* and *PbrPP2C49*), three were pollen-specific (*PbrPP2C9*, *PbrPP2C50,* and *PbrPP2C62*), and one gene exhibited sepal-specific expression (*PbrPP2C69*) (Figure 5). This analysis has identified candidate *PbrPP2C* genes that may play specialized roles in different organs’ development.

To further verify the role of PP2C-mediated stress response, we analyzed the transcriptome of pear under biotic/abiotic stresses and pathogen treatments based on unpublished and published data of our laboratory (Li et al., 2016; Yang and Huang, 2018). The findings of the heatmaps showed that a large number of *PbrPP2C* genes responded to stress, and *PbrPP2C* genes showed variation in their expression pattern among different treatments (Supplementary Figure S2). In addition, most of *PbrPP2Cs* from subgroup A were also differentially upregulated by exposure to cold, drought, salt, and pathogen treatments, such as *PbrPP2C1*, *PbrPP2C4*, *PbrPP2C7*, *PbrPP2C11*, *PbrPP2C17,* and *PbrPP2C18* (Supplementary Figure S2). The diversity in the expression profiling of *PbrPP2C* genes may suggest that these *PbrPP2C* genes were stress-responsive.

### qRT-PCR Analysis of *Pbr*PP2C

**FIGURE 6:**
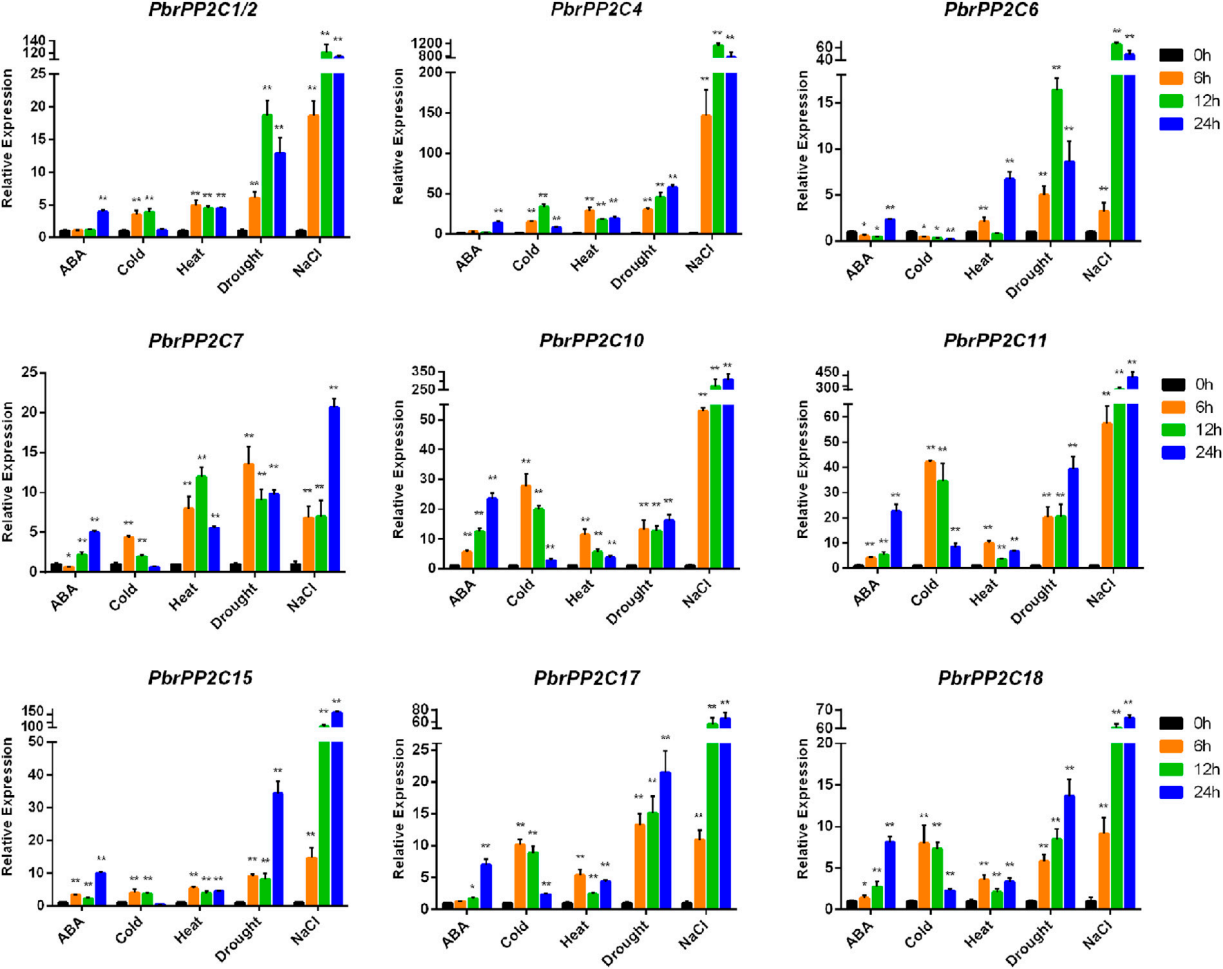
QRT-PCR analysis of subgroup A *PbrPP2C* genes under exposure to heat, cold, drought, NaCl, and ABA treatments in pear seedlings. Standard errors and ANOVA were calculated by applying Student’s t-test. Single and double stars stand for the levels of significant difference at *p*-value < 0.05 and <0.01, respectively.

To explore *PbrPP2C* gene expression under different stress conditions and identify *PbrPP2C* genes important for improving tolerance, the seedlings were subjected to heat, cold, drought, NaCl, and ABA treatments. It has been verified that subgroup A *PP2Cs* in *Arabidopsis* and rice were transcriptionally upregulated under exogenous ABA treatment or abiotic stress that stimulates ABA biosynthesis (Xue et al., 2008; Singh et al., 2010). Nine *PbrPP2C* genes from subgroup A were selected to perform qRT-PCR at different time points after various treatments. QRT-PCR after exogenous ABA treatment indicated that four genes (*PbrPP2C10*, *PbrPP2C11*, *PbrPP2C15,* and *PbrPP2C18*) were upregulated at three different time points: 6, 12, and 24 h. Four more genes (*PbrPP2C1/2*, *PbrPP2C4*, *PbrPP2C6,* and *PbrPP2C17*) were upregulated more than 5-fold in 24 h after exogenous ABA treatment. In contrast, the expression of *PbrPP2C6* and *PbrPP2C7* decreased to less than half that of CK in 6 and 12 h, respectively (Figure 6). Nine *PbrPP2C* genes from subgroup A were upregulated in response to more than one treatment with abiotic stress (Figure 6). For example, the *PbrPP2C6* expression level increased more than 3-fold under exposure to heat, drought, and salt treatment, but exposure to cold repressed its expression (Figure 6). All nine genes exhibited strongly increased expression levels (from 10-fold to 400-fold greater than CK) in response to NaCl treatment (Figure 6). Some results were consistently consistent with the analysis of heatmaps (Supplementary Figure S2). Taken together, the expression characteristics of subgroup A *PbrPP2C* genes indicate that these nine genes respond to exogenous ABA and abiotic stress.

### Subcellular Localization of PbrPP2C Protein

**FIGURE 7:**
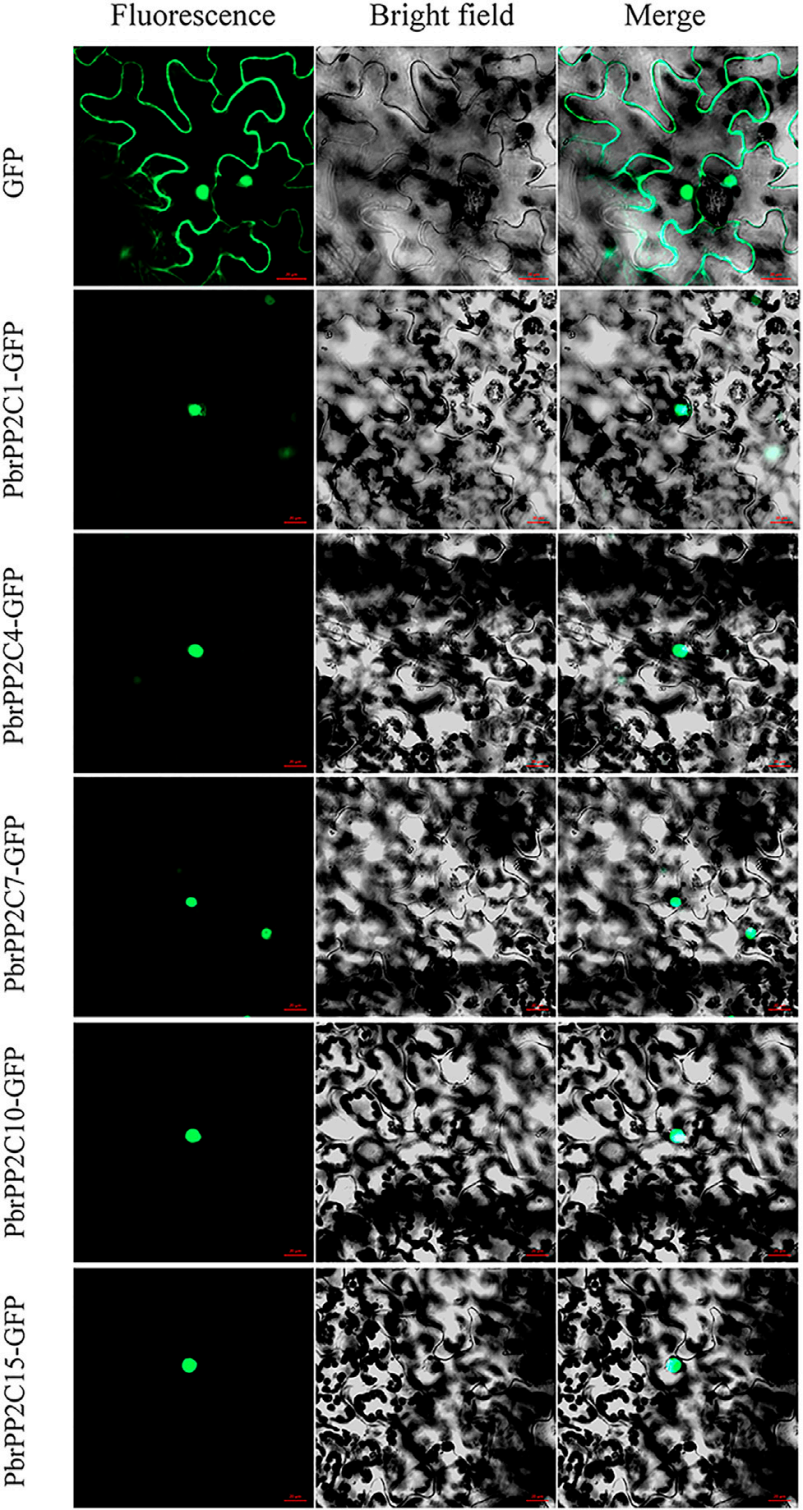
Subcellular localization of the fusion protein PbrPP2Cs-GFP in *N. benthamiana* leaves. Vector 35S-GFP was used as the control. Bar = 20 μm.

Previous research has shown that PbrPP2C proteins localize in the nucleus (Haider et al., 2019; Kim et al., 2012). To determine the subcellular localizations of PbrPP2C proteins, five *PbrPP2C* genes (*PbrPP2C1, PbrPP2C4, PbrPP2C7, PbrPP2C10,* and *PbrPP2C15*) were selected from each branch from subfamily A of the *PbrPP2C* family. PbrPP2Cs-GFP recombinant plasmids were introduced into *N. benthamiana* leaves. The fluorescence confirms the localization of five PbrPP2C-GFP fusion proteins in the nucleus (Figure 7). ABA receptor proteins, namely PYR/PYL/RCRA, are localized in both the cytoplasm and nucleus, despite the fact that interacting PP2C proteins are localized exclusively in the nucleus (Santiago et al., 2009; Kim et al., 2012). This observation suggests that PbrPP2C may play a transcriptional regulatory role in the nucleus.

## Discussion

The landscape of the *PP2C* genes family in the plant kingdom has been characterized in previous research studies, such as in *Arabidopsis* (Kerk et al., 2002), rice (Singh et al., 2010), maize (Wei and Pan 2014), banana (Hu et al., 2017), *Brachypodium distachyon* (Cao et al., 2016), *Brassica rapa* (Khan et al., 2019), and *Gossypium hirsutum* (Shazadee et al., 2019). However, the *PP2C* gene family has not been studied widely in the Rosaceae family. The current study provides a comprehensive analysis of the *PP2C* gene family in eight Rosaceae species, including gene identification, phylogenetic relationships, chromosomal localizations, and evolutionary analysis. 719 *PP2Cs* were identified, ranging from 65 genes in strawberry to 128 in apple. The genes exhibited widespread and uneven distribution across Rosaceae chromosomes (Figure 3). In Chinese white pear, 118 *PbrPP2C* genes were further categorized into twelve subgroups A–L and one unclassified subgroup according to phylogenetic and evolutionary analysis (Figure 1). This classification of *PP2C* genes was consistent with previous studies, such as those conducted on *Arabidopsis* (Kerk et al., 2002) and rice (Singh et al., 2010). *PbrPP2C* genes were found to be expressed in specific organs, providing strong evidence of specialized function. At least eight *AtPP2C* genes from subgroup A were key factors in the ABA signaling network (Hirayama and Umezawa, 2010). *AP2C1* of subgroup B was involved in suppressing MAPK activates in response to wounding or pathogen stresses (Schweighofer et al., 2007). *POL* and *PLL1* of subgroup C were involved in regulating flower development and maintain stem cell polarity (Song et al., 2008; Gagne and Clark, 2010). *PP2C* of subgroup E and *AtPP2C6-6* were involved in modulating stomata signaling (Servet et al., 2008). *WIN2* of subgroup F was involved in inducing the stress response (Lee et al., 2008). Overall, *PP2C* genes of the same subgroup have similar specialized biological functions, although the functions of many *PP2C* subgroups are still unclear.

Gene duplication is the predominant driving force for broad expansion of the gene family, which could obtain new functions and evolutionary processes (Qiao et al., 2019). The different types of gene duplications, including WGD, TD, PD, TRD, and DSD (Doerks et al., 2002; Moore and Purugganan, 2003), contribute differently to the expansion of gene families (Freeling, 2009). In the genome and genetic evolutionary system, WGD is the main driving force of new functions and features of eukaryotic genome evolution (Friedman and Hughes, 2001; Moore and Purugganan, 2003). For instance, the *BES1* and *GhERF* subfamily *B3* group gene families in cotton were expanded primarily though segmental or WGD duplication events (Liu et al., 2018; Lu et al., 2021). *F-box* and heat-shock transcription factor families in pear were expanded primarily through WGD and DSD (Qiao et al., 2015; Wang et al., 2016). In our study, we demonstrated WGD replication events in eight Rosaceae species (Figure 3). We also show that WGD and DSD were the driving forces for the expansion of the *PP2C* gene family in Chinese white pear, European pear, and apple (Figure 2). Finally, we found that most *Ka*/*Ks* ratios of *PP2C* gene pairs were less than one, suggesting that these genes have experienced strong purifying selection.

Surrounded by various stress factors, such as ABA, drought, salt, heat, cold, and phytohormonal stresses, are the major limiting factors of plant development and agricultural crop production, and the role of ABA signaling in stress adaptation and stress resistance mechanisms has been well documented (Sugimoto et al., 2014; Singh et al., 2016). Group A of *PP2Cs* comprises of negative regulators of ABA signaling by PYL intracellular receptors (Antoni et al., 2012). In *Arabidopsis*, at least six genes of group A *PP2C* (*ABI1*, *ABI2*, *HAB1*, *HAB1*, *HAB2*, *PP2CA,* and *AHG1*) resulted in increasing the ABA sensitivity under various stresses, indicating the diverse outcome in ABA signaling (Merlot et al., 2001; Tähtiharju and Palva, 2001; Umezawa et al., 2009). In *Fagus sylvatica*, two genes of group A *PP2C* (*FsPP2C1* and *FsPP2C2*) resulted in influencing ABA sensitivity and tolerance of abiotic stress in seeds (Gonzalez-Garcia et al., 2003; Reyes et al., 2006). In maize, *ZmPP2C-A10* of subgroup A of *PP2C* was confirmed for its negative regulation in drought stress (Xiang et al., 2017). Although *PP2C* genes in subgroup A have been demonstrated to play key roles in various stress conditions in some species, the role of the key components of ABA signaling against *PP2C* genes is mainly obscure in pear. In the present study, nine *PbrPP2C* genes from subgroup A exhibited substantial transcriptional variations when confronted by heat, cold, drought, and NaCl challenges and in response to ABA treatment, indicating their regulatory role in stress tolerance. Gene expression levels exhibited, especially, strong response to salt stress (Figure 6). Therefore, the study reveals potential functions of *PP2C* genes in a commercially important angiosperm family. However, validation of the individual gene product’s function at the molecular level remains an important step in understanding *PP2C* genes in the Rosacaea family in future.

## 5. Conclusion

In this study, a total of 719 *PP2C* gene family members were first identified in eight Rosaceae species. The *PP2C* gene pairs of Rosaceae species might evolve undergoing strong purifying selection. The 118 *PbrPP2C* genes of Chinese white pear were classified into twelve subgroups according to the phylogenetic relationship gene structure and protein motif pattern. Moreover, qRT-PCR revealed nine candidate genes from subgroup A which might have participated in the plant stress tolerance particularly to ABA, heat, cold, drought, and NaCl stress. Subcellular localization analysis proved the functionality of five *PbrPP2C* genes from each branch of subfamily A in the nucleus. Consequently, our findings provide a foundation for the potential function of *PbrPP2C* genes under various stress conditions.

## Data Availability

The original contributions presented in the study are included in the article/Supplementary Material; further inquiries can be directed to the corresponding authors.

## Abbreviations

PK, protein kinase; PP, protein phosphatase; Ser, serine; Thr, threonine; Tyr, tyrosine; PTP, protein threonine phosphatase; STP, serine/threonine phosphatase; DSPTP, dual-specificity phosphatase; PPP, phosphor-protein phosphatase; PPM, the Mg2+- or Mn2+-dependent protein phosphatase; PP2C, type 2C protein phosphatase; HMM, hidden Markov mode; WGD, Whole-genome duplication; TD, tandem duplication; TRD, transposed duplication; PD, proximal duplication; DSD, dispersed duplication; ML, maximum likelihood; PGDD, llant genome duplication database; GDR, Genome Database for Rosaceae; RPKM, reads per kilobase of exon model per million mapped reads; Ka, non-synonymous substitution rates; Ks, synonymous substitution rates.
