# Supplementary figures and images for: Genome-wide Identification and Evolution of the PP2C Gene Family in Eight Rosaceae Species and Expression Analysis Under Stress in Pyrus bretschneideri
Source: Front Genet. 2021 Nov 11;12:770014. doi: 10.3389/fgene.2021.770014 (PMC8632025; doi:10.3389/fgene.2021.770014)

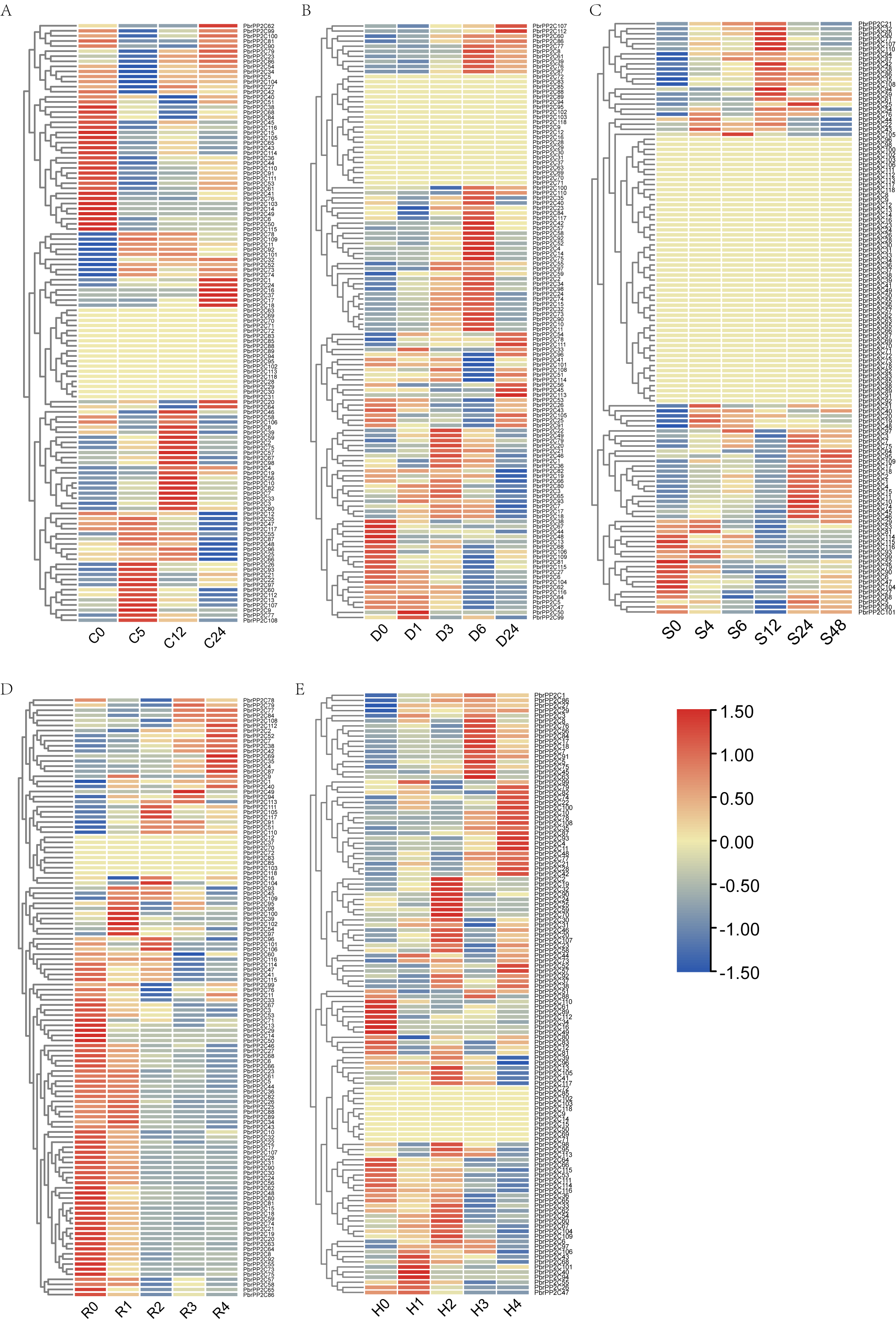

Supplement: Supplementary file 1 [file Image2.TIF]

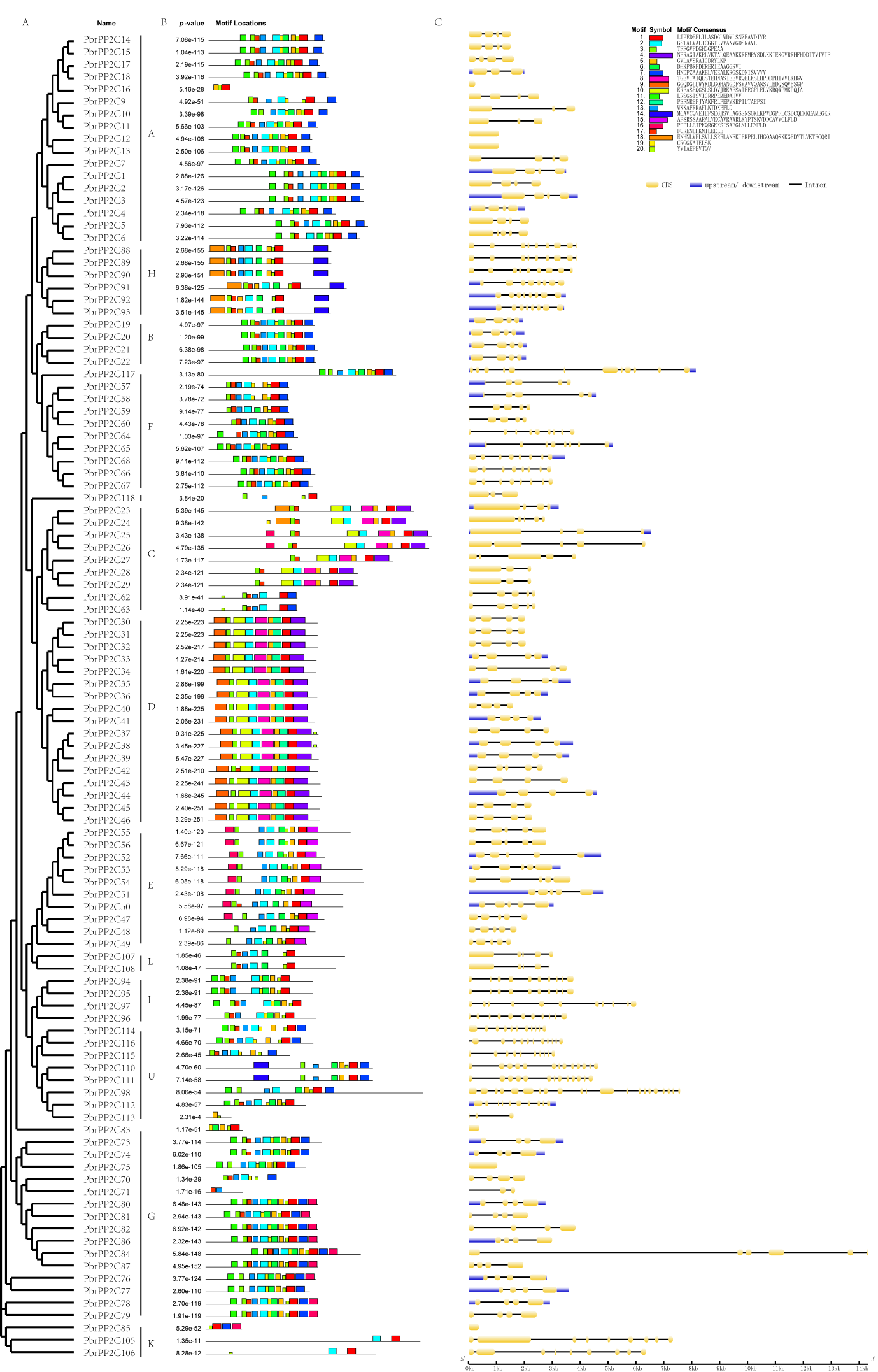

Supplement: Supplementary file 2 [file Image1.TIF]
